# Papilledema: Point-of-Care Ultrasound Diagnosis in the Emergency Department

**DOI:** 10.5811/cpcem.2018.1.36369

**Published:** 2018-03-14

**Authors:** Joseph R. Sinnott, Mohammad R. Mohebbi, Timothy Koboldt

**Affiliations:** University of Missouri School of Medicine, Department of Emergency Medicine, Columbia, Missouri

## Abstract

Point-of-care ultrasound (POCUS) has the potential to diagnose papilledema, a sign of increased intracranial pressure, through optic disc elevation as well as optic nerve sheath diameter measurements. Idiopathic intracranial hypertension (IIH) is a syndrome resulting in increased intracranial pressure. We present a case of IIH where the emergency physician diagnosed papilledema by POCUS via presence of both optic disc elevation and a widened optic nerve sheath diameter.

## INTRODUCTION

Idiopathic intracranial hypertension (IIH) is a syndrome of elevated intracranial pressure of unknown etiology that typically presents in females of childbearing age.^2^ IIH is a diagnosis of exclusion and thus requires a thorough investigation into other potential causes of increased intracranial pressure.^2^ Neurodiagnostic studies are often unremarkable aside from indicating increased cerebrospinal fluid pressure on lumbar puncture.^2^ Papilledema and loss of visual function are potential signs and symptoms, respectively, of IIH.^2^ Misdiagnosis and/or improper treatment of IIH can lead to optic atrophy and irreversible visual loss.^2^ In this report we describe a case of IIH that presented with headache and vision changes. The presence of both optic disc elevation and widened optic nerve sheath diameter in the setting of IIH are testament to the novelty of this case. Papilledema was visualized by point-of-care ultrasound (POCUS).

## CASE REPORT

A 27-year-old African-American female with a past medical history of obesity (body mass index: 39) presented to the emergency department (ED) with complaints of headache and vision changes. The patient reported an approximate two-week history of what she described as a throbbing bitemporal headache. She noted partial relief of her headache with alternating courses of acetaminophen and ibuprofen every four hours. Additionally, she developed intermittent painless vision changes one week prior to the ED visit. She reported that her vision would transiently go dark bilaterally upon opening her eyes. These episodes would self-resolve after approximately one minute. One day prior to her ED visit, the patient endorsed complete painless loss of vision in her left eye, after what she described as a black screen coming over her field of vision while driving. She had no associated symptoms of eye pain, trauma, fevers, neck stiffness, numbness, tingling, weakness, or facial droop.

The patient’s vital signs on presentation included temperature 36.7° Celsius, blood pressure 124/78, heart rate 70 beats per minute, and oxygen saturation of 98% on room air. On ophthalmologic examination, pupils were equal and extraocular movements were intact. The patient exhibited 20/20 visual acuity in the right eye. Visual acuity in the left eye revealed loss of peripheral vision and minimal light perception centrally. There was also an afferent pupillary defect of the left eye on swinging light reflex. Fundoscopy in the ED was limited by photophobia, and the optic discs could not be well visualized on undilated examination. Pressure in the left eye and right eye were measured at 23.95 millimeter of mercury (mmHg) and 24.90 mmHg via tonopen, respectively. Non-contrast head computerized tomography (CT) revealed a hyperdense lesion within the posterior sella, which was unchanged from 17 months prior. Otherwise, CT revealed no acute intracranial findings.

In addition to performing a head CT, point-of-care ultrasound (POCUS) was performed in the ED. POCUS of the left eye revealed elevation of the optic disc into the vitreous cavity, consistent with papilledema (Image; Video).^3^ Ultrasound also revealed an enlarged optic nerve sheath diameter of 7.4 millimeters (mm) (Image). Concern for papilledema prompted emergent ophthalmology and neurology consultation for further assessment. Ophthalmology assessment revealed Grade 3–4 optic disc edema bilaterally, with probable optic neuropathy of the left eye secondary to optic disc edema.

The patient was admitted to neurology for further workup and management. Magnetic resonance imaging (MRI) of the brain obtained upon admission revealed no acute findings or masses that could account for increased intracranial pressure. MRI did demonstrate a posterior pituitary adenoma measuring 10 x 8 mm, but it was not compressing on the optic chiasm or optic nerves. MRI of the brain and sella pre- and post-gadolinium confirmed a pituitary lesion that was unchanged in size dating back to 17 months prior. Laboratory tests assessing pituitary function, including thyroid-stimulating hormone, free thyroxine, and prolactin, resulted within normal limits. CT venogram of the brain was also completed upon admission. Results revealed a hypoplastic right transverse sinus without evidence of sinus thrombosis.

Of importance, lumbar puncture revealed an increased opening pressure of 51 cm H_2_O, with a closing pressure of eight cm H_2_O. Lack of space-occupying lesions on imaging with associated increased opening pressure on lumbar puncture suggested a diagnosis of IIH.^2^ The patient’s headache improved following a large-volume lumbar puncture; however, she continued to have visual field defects in the left eye. The patient was started on acetazolamide 500 milligrams every six hours. Neurosurgery was consulted, and she underwent ventriculoperitoneal shunt placement. Her headache subsequently resolved, with modest improvement in her vision.

**CPC-EM Capsule**

What do we already know about this clinical entity?

Idiopathic intracranial hypertension is a syndrome resulting in increased intracranial pressure. Papilledema can be visualized via point-of-care ultrasound (POCUS).

What makes this presentation of disease reportable?

This case was significant for presence of both optic disc elevation and widened optic nerve sheath diameter in a patient with idiopathic intracranial hypertension who presented with headache and vision changes.

What is the major learning point?

Diagnosis of papilledema by POCUS was an effective adjunct in the eventual diagnosis and management of idiopathic intracranial hypertension.

How might this improve emergency medicine practice?

POCUS has the potential to diagnose papilledema in those suspected of having increased intracranial pressure.

She was discharged home seven days after admission, with scheduled follow-up appointments with neurosurgery, neurology, and ophthalmology. Upon follow-up with ophthalmology, there was noted improvement of papilledema in both eyes, but with clinical evidence of optic neuropathy and optic nerve atrophy in the left eye.

## DISCUSSION

IIH is a syndrome that results in elevated intracranial pressure. The incidence of this syndrome is more common in women who are obese and of childbearing age.^2^ Additionally, it is a diagnosis of exclusion; thus, other etiologies of increased intracranial pressure must be eliminated via history, physical exam, and neurodiagnostic studies.^2^

Symptoms of IIH in order of decreasing frequency include headache, transient visual disturbances, tinnitus, photophobia, retrobulbar pain, and visual loss.^2^ Headache, commonly described as pulsatile, is commonly the presenting symptom, as was the case in this patient.^2^ Visual disturbances can include monocular or binocular transient episodes of blurred vision.^2^ These transient episodes of visual disturbance are postulated to be secondary to temporary ischemia of the optic nerve due to increased intracranial pressure.^2^ Similarly, our patient initially had complaints of transient visual field obscurity bilaterally. However, it is postulated that these transient visual field changes are not associated with poor visual field outcomes.^2^ Papilledema is a manifestation of increased intracranial pressure in those with IIH.^2^ Papilledema in cases of IIH is typically bilateral, but can be unilateral.^2^ Papilledema can result in permanent vision loss and optic atrophy if not diagnosed and treated in a timely manner.^2^

The patient’s case demonstrated the use of POCUS for early diagnosis of papilledema at bedside. The presence of optic disc elevation and enlarged optic nerve thickness, in the setting of IIH, points to the novelty of this case. Prior studies have illustrated that optic disc elevation, with a minimum disc height of 0.6 mm, obtained via ultrasound can be 82% sensitive and 76% specific for papilledema.^3^ Disc height via ultrasound can be acquired by measuring the distance between the anterior peak of the disc and its intersection to the posterior surface of the globe. Optic nerve sheath diameter measurements obtained via ultrasonography can also be a simple and sensitive indicator of increased intracranial pressure.^4^

Using invasive intracranial monitoring as a reference standard, previous studies have revealed that an optic nerve sheath diameter of greater than five mm, measured three mm posterior to the orbit, can be a sensitive (88%) and specific (93%) marker for elevated intracranial pressure of greater than 20 cm H_2_O.^5^,^6^ POCUS for our patient demonstrated a left optic nerve sheath diameter of 7.4 mm, measured three mm posterior to the orbit, suggesting increased intracranial pressure (Image). This was confirmed by lumbar puncture upon admission, which revealed an opening pressure of 51 cm H_2_O.

Evidence of papilledema on ultrasound and elevated opening pressure on lumbar puncture, with unremarkable imaging studies, led to the diagnosis of IIH. This was further supported by improvement of the patient’s headache and visual changes following appropriate treatment with acetazolamide, large volume cerebrospinal fluid drainage, and ventriculoperitoneal shunt placement. However, the patient suffered from residual visual field deficits in the left eye secondary to papilledema and optic nerve atrophy. This points towards the need for early detection of papilledema, potentially via ocular ultrasonography, in order to initiate timely consultations and treatment regimens.

## CONCLUSION

Emergency physicians may find potential benefit in the use of POCUS to diagnose papilledema in those patients suspected of having increased intracranial pressure. Optic disc elevation of greater than 0.6 mm, in addition to optic nerve sheath diameter of greater than five mm, have been shown to be correlated with papilledema.^3^,^5^,^6^ Visualization of papilledema by POCUS in the ED may serve as an effective adjunct or alternative to traditional funduscopic examinations in the diagnosis and management of IIH.

Documented patient informed consent and/or Institutional Review Board approval has been obtained and filed for publication of this case report.

## Supplementary Information

Video Left ocular ultrasound demonstrating optic disc elevation (white arrow).

## Figures and Tables

**Image:**
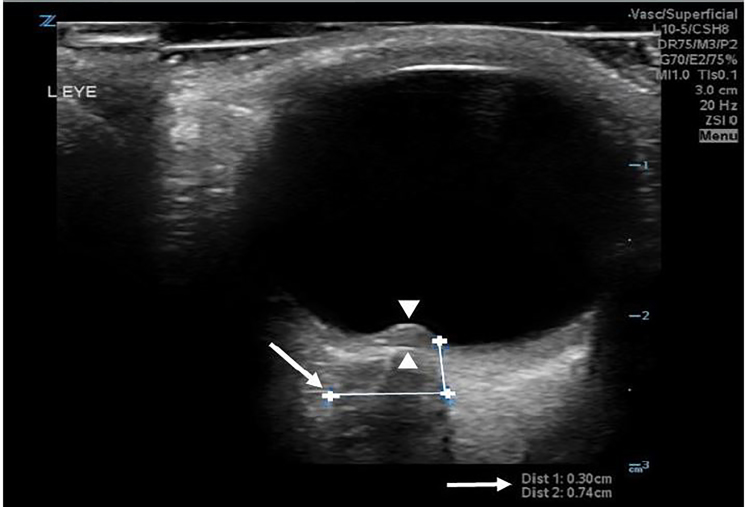
Left ocular ultrasound demonstrating optic disc elevation into the vitreous cavity (white arrow heads), as well as optic nerve sheath diameter measurements (white arrows)
