# Supplementary material for: Papilledema: Point-of-Care Ultrasound Diagnosis in the Emergency Department
Source: Clin Pract Cases Emerg Med. 2018 Mar 14;2(2):125–7. doi: 10.5811/cpcem.2018.1.36369 (PMC5965109; doi:10.5811/cpcem.2018.1.36369)
Supplement: Video — Left ocular ultrasound demonstrating optic disc elevation (white arrow). [file cpcem-02-125-s001.docx]

Video:

Left ocular ultrasound demonstrating optic disc elevation (white arrow).
